# A case of cardiac langerhans histiocytosis

**DOI:** 10.1186/1532-429X-11-S1-T7

**Published:** 2009-01-28

**Authors:** Ashley Simmons, Thomas Rosamond

**Affiliations:** University of Kansas Medical Center, Kansas City, KS USA

**Keywords:** Histiocytosis, Obstructive Coronary Artery Disease, Viral Myocarditis, Eosinophilic Granuloma, Normal Left Ventricular Ejection Fraction

## Introduction

Langerhans histiocytosis (eosinophilic granuloma) is a disease characterized by proliferation of specialized bone marrow-derived Lnagerhans cells and mature eosinophils. The disease is rare and typically found in children. There is debate over whether the disease is neoplastic or reactive. To date, there have been no cases of Langerhans cell histiocytosis involving the myocardium.

## Case report

A 44 year old female with a past medical history of hypertension presented to the emergency department with complaints of chest pain. Her troponin was elevated and peaked at 3.76. She underwent cardiac catheterization which showed a normal left ventricular ejection fraction and no significant obstructive coronary artery disease. Chest CT showed nodular and cystic opacities within the upper lobe suggestive of Langerhans cell histiocytosis. There was no evidence of pulmonary embolism or aortic dissection, no lymphadenopathy. Cardiac MRI was performed and showed a focal area of increased T1 signal intensity, decreased perfusion and heterogeneous transmural delayed hyperenhancement in the posterior basal segment of the left ventricle. A diagnosis of fibroxanthomatous infiltrative myocardial disease due to Langerhans Histiocytosis was made on the basis of cardiac MR images and CT chest images. Transbronchial biopsy and broncho-alveolar lavage showed no evidence of histiocytosis. The differential diagnosis remains focal eosinophilic granuloma versus viral myocarditis.

## Conclusion

Cardiac MRI can help with the differential diagnosis of infiltrative myocardial diseases with delayed hyperenhancement techniques.

